# Effect of Initial Powders on Properties of FeAlSi Intermetallics

**DOI:** 10.3390/ma12182846

**Published:** 2019-09-04

**Authors:** Jaroslav Čech, Petr Haušild, Miroslav Karlík, Václav Bouček, Kateřina Nová, Filip Průša, Pavel Novák, Jaromír Kopeček

**Affiliations:** 1 Department of Materials, Faculty of Nuclear Sciences and Physical Engineering, Czech Technical University in Prague, 12000 Prague, Czech Republic (P.H.) (M.K.) (V.B.); 2 Department of Metals and Corrosion Engineering, University of Chemistry and Technology, 16628 Prague, Czech Republic (K.N.) (F.P.) (P.N.); 3 Institute of Physics of the Czech Academy of Sciences, 18221 Prague, Czech Republic

**Keywords:** FeAlSi, intermetallic alloys, mechanical alloying, spark plasma sintering, microstructure, nanoindentation, mechanical properties

## Abstract

FeAlSi intermetallics are materials with promising high-temperature mechanical properties and oxidation resistance. Nevertheless, their production by standard metallurgical processes is complicated. In this study, preparation of powders by mechanical alloying and properties of the samples compacted by spark plasma sintering was studied. Various initial feedstock materials were mixed to prepare the material with the same chemical composition. Time of mechanical alloying leading to complete homogenization of powders was estimated based on the microstructure observations, results of XRD and indentation tests. Microstructure, phase composition, hardness and fracture toughness of sintered samples was studied and compared with the properties of powders before the sintering process. It was found that independently of initial feedstock powder, the resulting phase composition was the same (Fe_3_Si + FeSi). The combination of hard initial powders required the longest milling time, but it led to the highest values of fracture toughness.

## 1. Introduction

Intermetallic materials are used in a wide range of applications for some of their irreplaceable physical and mechanical properties [1]. Iron aluminides were one of the most studied materials especially due to their low weight and good oxidation resistance compared to austenitic stainless steels or nickel-based superalloys [2]. Improvement of Fe_3_Al and FeAl based iron aluminides was achieved by alloying by Cr, Nb, Zr, Co, Ce, Ti, etc. (e.g., [3,4,5,6,7]). Much less attention was focused on the ternary system Fe-Al-Si because of the brittle behavior of these alloys. In the previous studies, it was found that the system FeAl20Si20 (wt.%) has excellent high temperature oxidation and sulphidation resistance [8,9]. Therefore, they are the potential candidates for replacement of stainless steels and nickel superalloys, which can reduce the amount of the necessary critical raw materials used for their production [10].

The production of these alloys by standard metallurgical processes such as casting followed by hot/cold rolling is practically not possible because of their brittleness. For this reason, mechanical alloying (MA) was used to prepare the powders suitable for further sintering. Mechanical alloying is the process which starts from blended powder mixtures which are exposed to severe deformation in a high-energy ball charge. During mechanical alloying, consisting of repeated cold welding, fracturing and rewelding, the powder particles become homogeneous [11,12]. Mechanical alloying can lead to formation of very fine-grained microstructure with metastable phase composition which cannot be reached by other methods. The conditions of mechanical alloying process, especially the ball-to-powder ratio, rotational speed and lubrication, have a crucial effect on the structure and phase composition of the product. The use of a lubricant minimizes the adhesion of the powder to the walls of the milling jar and balls and hence it reduces the contamination caused by the adhesion wear of these parts. On the other hand, it decreases friction force and lowers the temperature of the milled powder [13]. The ball-to-powder ratio affects the required milling duration strongly. The higher is the ratio, the higher energy is supplied to the powder and thus the process is more efficient and faster [14]. Growing ball-to-powder ratio also increases the micro-strain and decreases the grain size of the product [14]. The rotational velocity also affects the required duration of mechanical alloying and also the morphology of the particles. In the case of brittle materials, higher rotational velocity produces finer powders. On the other hand, in the case of plastic materials it promotes flattening and cold welding of particles [15,16].

Ultra-high energy mechanical alloying was developed in the previous works [13,17]. It is based on high ball-to-powder ratio with large balls leading to collisions with high kinetic energy. Milling without any lubricant increases the friction forces and consequently the local temperature, which leads to or accelerates thermally activated reactions. As a consequence, the process of powder homogenization is faster than in the case of commonly used conditions of mechanical alloying.

In order to prevent grain coarsening or undesirable phase changes, the powder consolidation must be fast. For this reason, spark plasma sintering (SPS) was chosen as a sintering method. During SPS [18] the powder is pressed between two electrodes and high amperage electric current is applied. Joule heat and electric discharges rapidly heat the powder and consolidate it into the final sample. As it uses high sintering rates, it prevents the growth of fine-grained microstructure, which can be beneficial for mechanical properties of the material (especially the hardness and the fracture toughness which is very important in the case of brittle intermetallic alloys).

Kinetics of the mechanical alloying depends on several factors, such as batch size, powder to ball mass ratio, rotational speed, lubrication, milling time or initial powder composition [11]. This paper focuses on the ternary alloy FeAlSi with the (optimal) composition FeAl20Si20. The effect of the other compositions (various ratio of Al and Si) was studied in previous papers (e.g., [8,9,17,19]). It was found that changes in the ratio of Al and Si can affect final phase composition (phases FeAl, Fe_3_Si, Fe_2_Al_5_, FeSi, Fe_3_Al_2_Si_3_, FeAl_2_Si in different amounts were identified for different compositions), oxidation resistance and/or mechanical properties (hardness). In this study, the effect of the initial powders on the process of mechanical alloying of FeAl20Si20 alloy was investigated. The aim of this paper is to improve the processing method of this alloy. Structural and mechanical properties of the powders and compacted materials prepared by SPS technique are examined and compared.

## 2. Materials and Methods

### 2.1. Materials Preparation

**Figure 1:**
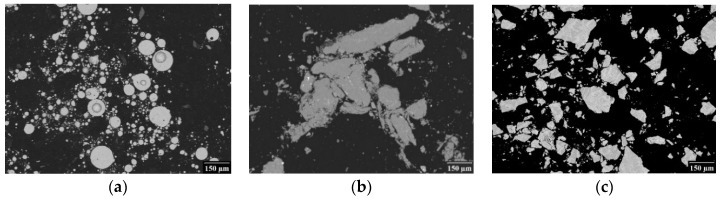
Microstructure of pre-alloyed powders (SEM BSE micrographs): (**a**) FeAl25, (**b**) AlSi30, (**c**) FeSi25.

Compacts of the final composition FeAl20Si20 were prepared from different initial feedstock materials. The initial composition FeAl20Si20 was chosen on the basis of the previous works, where the excellent high-temperature oxidation resistance and promising mechanical properties were identified for this alloy [9]. A given number of prerequisites were mixed in required ratios to obtain desired final composition of 60 wt.% of Fe, 20 wt.% of Al and 20 wt.% of Si. As an initial input powders pure Fe (Strem Chemicals, Newburyport, MA, USA, ~7 µm, purity 99.9%), Al (Alfa Aesar, Haverhill, MA, USA, ~44 µm, purity 99.5%), Si (Strem Chemicals, ~44 µm, purity 99.7%) and pre-alloyed AlSi30 (phase composition: Fe, Al and Si, particle size < 400 µm), FeAl25 (phase composition: Fe_3_Al and FeAl, particle size < 150 µm), FeSi25 (phase composition: Fe_3_Si and FeSi, particle size < 200 µm) were used. Scanning electron microscope (SEM) backscattered electron (BSE) images of pre-alloyed powders are presented in Figure 1. Four mixtures were prepared:

- Fe + Al + Si (denoted Fe_Al_Si)
- FeAl25 + Si (denoted FeAl_Si)
- Fe + AlSi30 + Si (denoted Fe_AlSi)
- FeSi25 + Al (denoted FeSi_Al)

Powders were mixed and mechanically alloyed in planetary ball mill PM 100 CM (Retsch, Haan, Germany) in argon atmosphere. Five grams of powder were milled by balls made of the same material as the mill-AISI 420 steel, the powder to ball mass ratio was 1:60 and rotational speed 400 rpm. Powders were milled for 1, 2, 4, 6 and 8 h and the evolution of their microstructure, phase composition and mechanical properties were studied.

Powders were compacted into discs with diameter of 20 mm by spark plasma sintering using HP D10 device (FCT Systeme GmbH, Rauenstein, Germany). Powders were heated to 1000 °C (heating rate 300 °C/min up to 900 °C and then 100 °C/min to 1000 °C to avoid overshoot of the temperature) and pressed to 48 MPa. The maximum temperature was held for 10 min followed by slow cooling rate 50 °C/min to avoid sample cracking due to thermal shocks.

### 2.2. Characterization Techniques

Microstructure, phase composition, hardness and Young’s modulus of the powders after different milling times and compacted samples after sintering were evaluated. The powders and sintered samples were embedded into carbon filler containing phenolic resin and metallographic cuts were prepared by standard procedures. The surface was finished by polishing in 0.04 µm colloidal silica. Microstructure was examined in backscattered electron (BSE) signal in scanning electron microscope (SEM) JEOL JSM 5510LV (JEOL, Tokyo, Japan) with iXRF 500 energy dispersive X-ray spectrometer (EDS) and transmission electron microscope (TEM) JEOL 2200FS equipped by JEOL large angle EDS. TEM characterization was first carried out on loose mechanically alloyed powders deposited on copper grid with holey carbon film. The sintered materials were very brittle and thus the first TEM samples were prepared by crushing of the SPS compacts in an agate mortar. Later, focused ion beam (FIB) milling (in Zeiss Auriga device) was used to prepare thin lamellae about 15 μm by 15 μm in size. Phase composition was studied by PANalytical X’Pert Pro X-Ray diffractometer (PANalytical, Almelo, The Netherlands) in Bragg-Brentano geometry with Cu cathode.

Instrumented indentation tests were performed to determine mechanical properties of the studied materials. Indentation force-penetration depth curves were evaluated by Oliver-Pharr method [20,21] and the hardness and Young’s modulus were calculated according to standard ISO 14,577 [22] on NHT^2^ nanoindentation tester (Anton Paar, Graz, Austria). At least 15 particles from every powder mixture were tested to obtain statistically representative value of hardness and Young’s modulus. As the hard powder particles are relatively small and embedded in the matrix with much lower hardness and Young’s modulus, nanoindentation tests with partial unloadings at 1, 2, 5 and 10 mN were performed to determine the effect of the surrounding matrix on the measured values of hardness and Young’s modulus of powder particles. It was found [23] that for the size of particles in the order of tens of micrometers, the values of hardness measured at the maximum load 2 mN (corresponding to the penetration depth of approximately 80 nm) were not affected by the surrounding resin and the scatter of the data was reasonably small. On the other hand, the measurements of Young’s modulus of hard particles in such softer surrounding matrix were affected even for the penetration depths lower than 0.25% of the size of the particles. This means that in the case of the studied materials, penetration depths should be less than 50 nm, which is not possible because it will not meet the conditions of the ISO standard [22]. As a consequence, measured values of Young’s modulus for powder particles were underestimated and they are not presented. Moreover, to avoid the effect of the particle size, which is changing with milling time [17], only the particles larger than 10 µm were tested to avoid the effect of the particle size in soft surrounding matrix on hardness measurements.

Based on this research, sintered samples were also tested at the maximum load of 2 mN in order to obtain the data for the same experimental conditions and to enable direct comparison of results.

**Figure 2:**
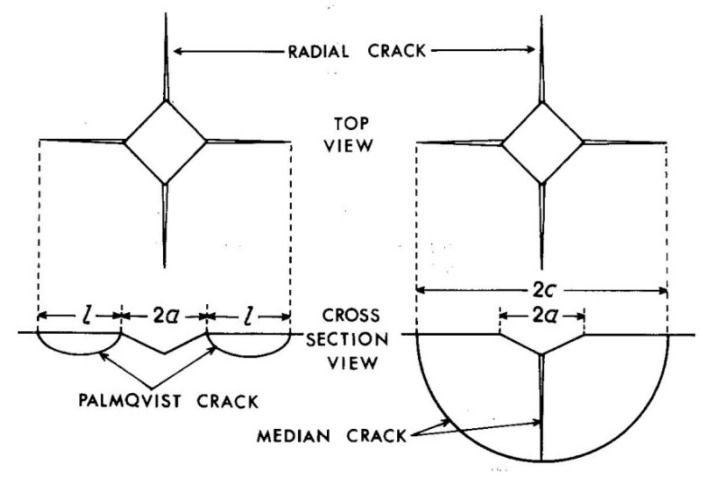
Geometry of the cracks created by the Vickers indentation [24].

To determine fracture toughness of the sintered materials, Vickers indentations with maximum load of 5 N were performed on MHT microindentation tester (Anton Paar, Graz, Austria) and the dimensions of radial cracks were measured (Figure 2). To evaluate the fracture toughness, model of Niihara [24] for Palmqvist cracks was employed. This model is based on the equation.

(1) KIC=0.035(la)−12Ha121ϕ(EϕH)25,

where *l* is the crack length, *a* is a half-diagonal of the indent, *H* is hardness, *E* stands for Young’s modulus and *ϕ* is the constraint factor (ratio of hardness and yield stress reaching the value of approximately 3).

## 3. Results

### 3.1. Powders

**Figure 3:**
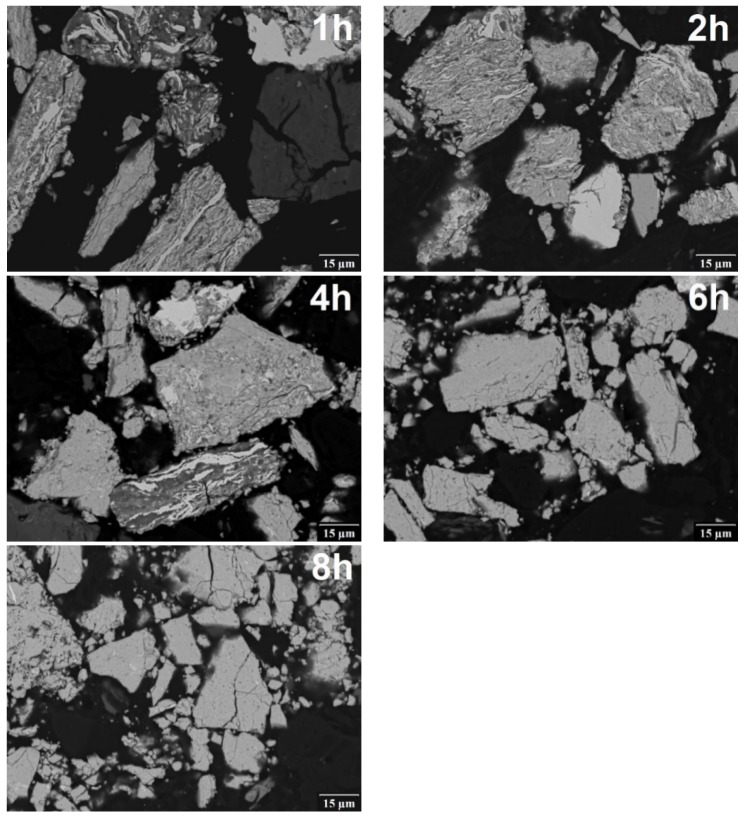
Typical example of the microstructure evolution with increasing time of mechanical alloying (Fe_AlSi powder mixture, SEM BSE micrographs).

**Figure 4:**
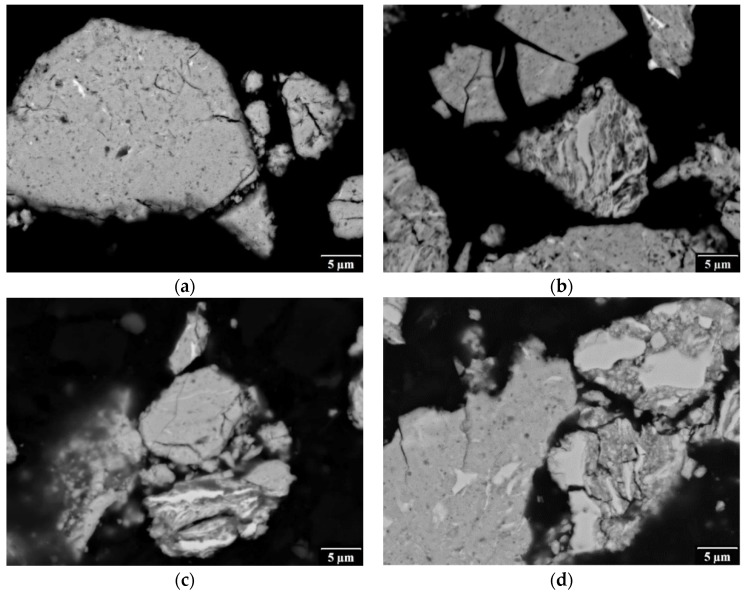
SEM BSE micrographs of powders after 4 h of mechanical alloying: (**a**) Fe_Al_Si, (**b**) FeAl_Si, (**c**) Fe_AlSi, (**d**) FeSi_Al.

Typical evolution of the microstructure of the powders is presented in Figure 3. At the beginning of the milling process, coarse lamellas of the initial powders in the individual powder particles and the unchanged particles of the initial powders were present. With increasing milling time (2 h), the lamellas got thinner, the occurrence of particles unaffected by the milling process was still rarer and first homogeneous particles with required chemical composition were observed. After four hours of milling (Figure 4), the homogeneous particles were present for all four powder mixtures. For the mixture of pure elements Fe_Al_Si, the homogeneous particles completely replaced heterogeneous particles. For the mixtures prepared from pre-alloyed powders, particles with lamellar structure could still be observed. Complete homogenization of Fe_AlSi and Fe_SiAl powders was observed after 6 h of milling. Only the powder Fe_AlSi was not completely homogenous and the particles with lamellar structure were observed. The complete homogenization of FeAl_Si powder occurred after 8 h of milling.

**Figure 5:**
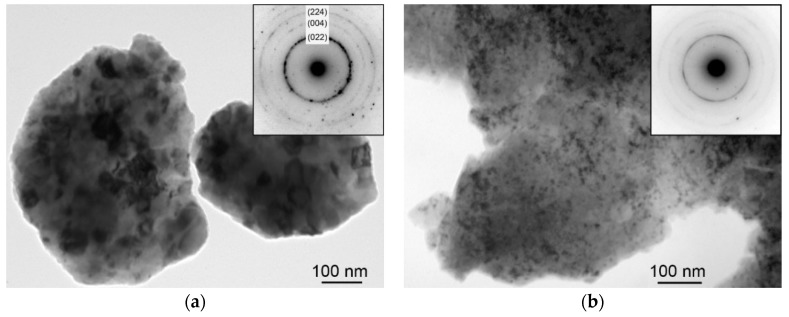

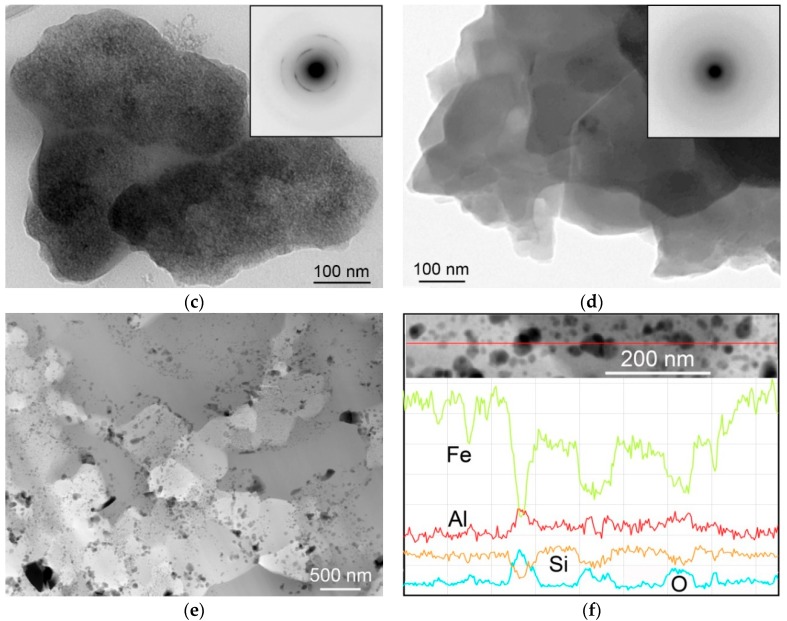
(**a**–**d**) Bright field TEM micrographs of: (**a**) nanocrystalline Fe_3_Si particles in the loose powder mixture from pure elements (Fe_Al_Si) after 8 h of mechanical alloying (related ring diffraction pattern is in the inset), (**b**–**d**) corresponding spark plasma sintering (SPS) compact crushed in agate mortar: (**b**) refined crystallites of the Fe_3_Si phase, (**c**) another (unidentified) phase with nanometric grains (**d**) amorphous phase, (**e**) STEM HAADF micrograph of a focused ion beam (FIB) lamella prepared from the same SPS compact, (**f**) STEM EDS line analysis across dark alumina particles in the micrograph (**e**).

From the low magnification TEM micrographs of the Fe_Al_Si powder after MA for 8 h (not presented here) it was obvious, that only particles under 5 μm in size had stuck on the carbon film. The chemical composition of these small particles obtained from TEM-EDS was in the range (wt.%) 55% to 66% of Fe, 10% to 15% of Al, 13% to 16% of Si, and 0.7% to 4% of O. Two typical Fe-rich particles, agglomerates of very small grains, 20 to 100 nm in size, are presented in Figure 5a. According to simulations performed using JEMS software (version 4.7830U2019b11) by Stadelmann [25], the diameters of the rings in the related electron diffraction pattern (inset in Figure 5a) correspond to the Fe_3_Si phase ($Fm\bar{3}m$, space group 225, a = 0.5655 nm).

**Figure 6:**
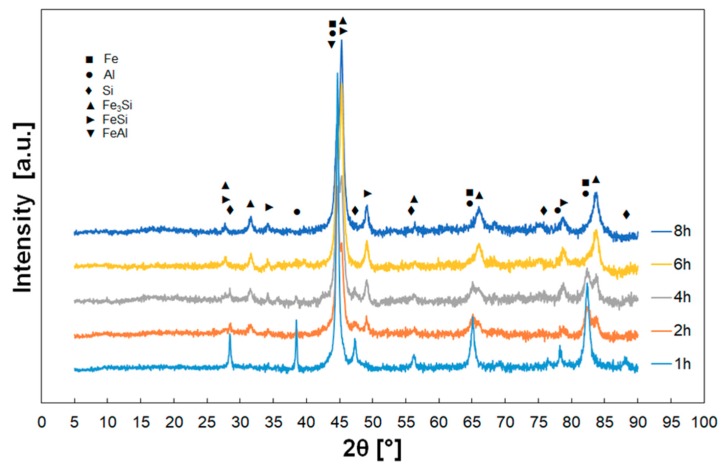
Typical example of the phase composition evolution with increasing time of mechanical alloying (Fe_AlSi powder mixture).

**Figure 7:**
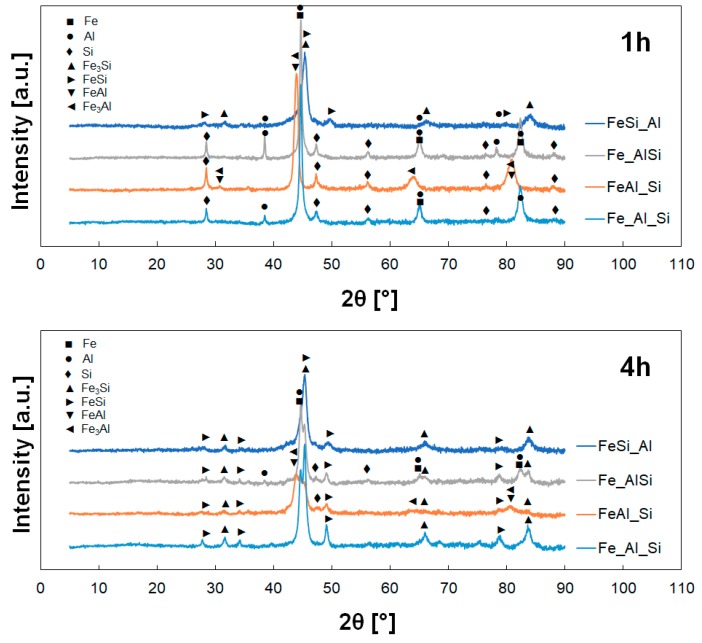

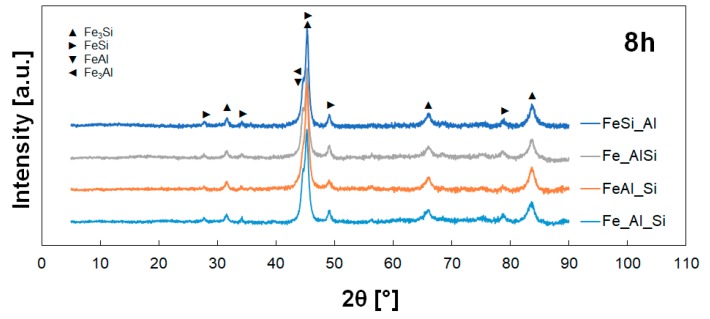
Phase composition of the powder mixtures in different stages of mechanical alloying.

Characteristic change of phase composition during mechanical alloying is shown in Figure 6 in the example of the Fe_AlSi powder mixture. At the beginning of the alloying process, the initial powders of the respective mixtures (i.e., Fe, Al, Si, and phases FeAl, Fe_3_Al, FeSi, Fe_3_Si in pre-alloyed powders) could be identified. With increasing milling time, the peaks of pure elements (Fe, Al and Si) disappeared and the peaks of the phases FeSi, Fe_3_Si and FeAl occurred and started to prevail for higher milling times. The change rate was different for different powder mixtures (Figure 7). At the end of the mechanical alloying process, only the phases FeSi, Fe_3_Si (supersaturated by Al) and small fraction of FeAl were present. The supersaturation of the phases FeSi and Fe_3_Si by Al atoms caused the increase of lattice parameters and shift of the peaks to the lower diffraction angles.

For Fe_Al_Si powder mixture from pure elements, the phases FeSi, Fe_3_Si and FeAl occurred after 2 h of milling and the peaks of original pure powders vanished after 4 h when the homogenization process was completed. The phases FeSi and Fe_3_Si, which formed the final powder mixture, started to occur after 4 h of milling for the mixture FeAl_Si. The phases of the initial powders (i.e., FeAl, Fe_3_Al and Si) were present even after 6 h of mechanical alloying and only after 8 h were not observed. The final phases (FeSi, Fe_3_Si and FeAl) for the mixture Fe_AlSi were firstly observed after 2 h of the mechanical alloying and the pure elements were lastly observed after 4 h of milling. The phases FeSi and Fe_3_Si in powder mixture FeSi_Al were present already from the beginning of milling because they formed the pre-alloyed powder; other phases disappeared after 4 h of milling.

**Figure 8:**
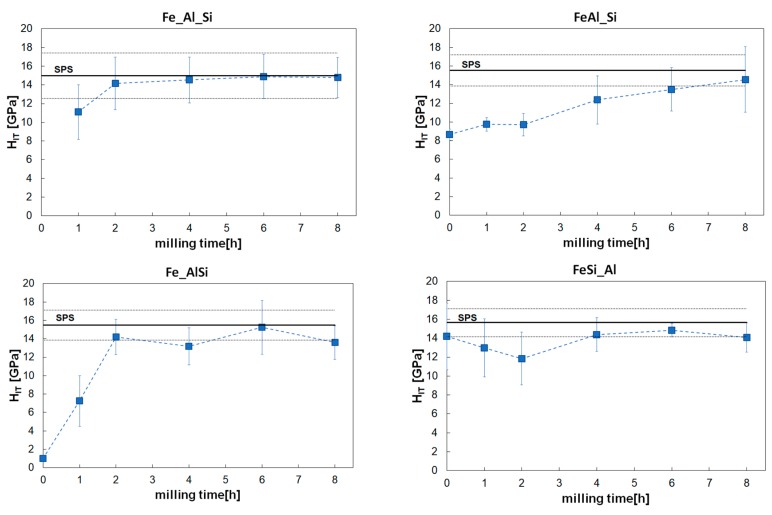
Hardness of the powder mixtures in different stages of mechanical alloying (solid horizontal line shows the value of the compacted sample measured at the maximum load of 2 mN, dotted lines represent the error bars).

Hardness values evaluated for maximum load 2 mN are presented in Figure 8. Values presented for 0 h correspond to initial pre-alloyed powders (i.e., FeAl25, AlSi30, FeSi25). Except FeSi_Al powder mixture, hardness increased at the beginning of the milling process. For Fe_Al_Si mixture, the significant increase ended after 2 h and the hardness stayed constant. For FeAl-Si mixture the increase was slower and more progressive up to the end of mechanical alloying at 8 h. Hardness of Fe_AlSi mixture started from the lowest values, but it increased very rapidly and it reaches the stable value after 2 h. On the other hand, mixture FeSi_Al started at very high hardness values (hard pre-alloyed powder FeSi25), hardness decreased to the minimum after 2 h after which it increased and it reached the constant value after 4 h. All the powder mixtures had hardness of approximately 15 GPa at the end of the mechanical alloying.

### 3.2. Compacted Materials

**Figure 9:**
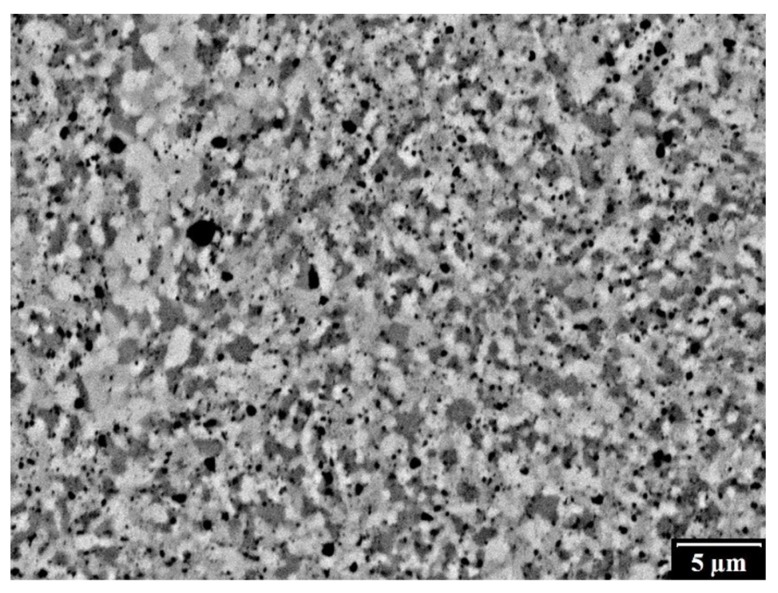
Microstructure of the compacted powder Fe_AlSi.

An example of the microstructure of the sintered sample by the SPS method is shown in Figure 9. It was homogeneous with a very low porosity (less than 0.1% for Fe_Al_Si sample [19,26], relative density nearly 100%, and fine microstructure (the grain size was around 1 µm for all four sintered samples—Table 1). There were not observed any significant differences between different initial powder mixtures as the powder mixtures used for SPS were of the same chemical and phase composition after MA.

From TEM analysis of the corresponding powder from the crushed Fe_Al_Si SPS compact it follows that SPS led to refining of the crystallites of the Fe_3_Si phase to nanometric size (Figure 5b), probably due to recrystallization (micrographs and diffraction patterns in Figure 5a,b are to scale). Isolated particles of other phases were also observed (Figure 5c). However, the crystallographic identification of these phases was not successful. Furthermore, amorphous nanoparticles were found (Figure 5d), formed possibly due to the local liquid phase sintering and rapid cooling of the compact. These amorphous phases may be at the origin of the high brittleness of the compact. According to EDS analysis, the chemical composition of the particles from the crushed compact was practically the same as of the loose MA powder.

From the scanning transmission electron microscopy (STEM) imaging of the FIB lamella prepared also from the Fe_Al_Si SPS compact using high-angle annular dark field detector (HAADF) it follows, that the grain size ranges from 100 nm up to 3 μm (Figure 5e). Many small particles were observed inside the grains and also on grain boundaries. The STEM HAADF signal is proportional to the scattering power (Z-contrast, where Z is the proton number); hence heavy elements appear bright, and holes and light particles appear dark. Therefore, the differences in the gray level of the grains in Figure 5e indicate a quite heterogeneous Fe concentration—grains appearing bright contain more iron, grains appearing dark contain more Al and Si. It can also be seen that the SPS compact includes a very high density of dark oxide particles, mostly 5 to 100 nm in size. These oxides are distributed inside of the majority of the grains and also on grain boundaries (Figure 5e). Figure 5f presents results of line STEM-EDS analysis across the particles, the red line in the inset indicating the path of the electron beam. From the plot it is obvious that majority of particles are oxides of aluminum. Therefore, argon flushing atmosphere during mechanical alloying did not prevent alumina formation. TEM analyses of the powders and compacts of the other three materials (FeAl_Si, Fe_AlSi, and FeSi_Al gave almost the same results, so they are not presented here).

**Figure 10:**
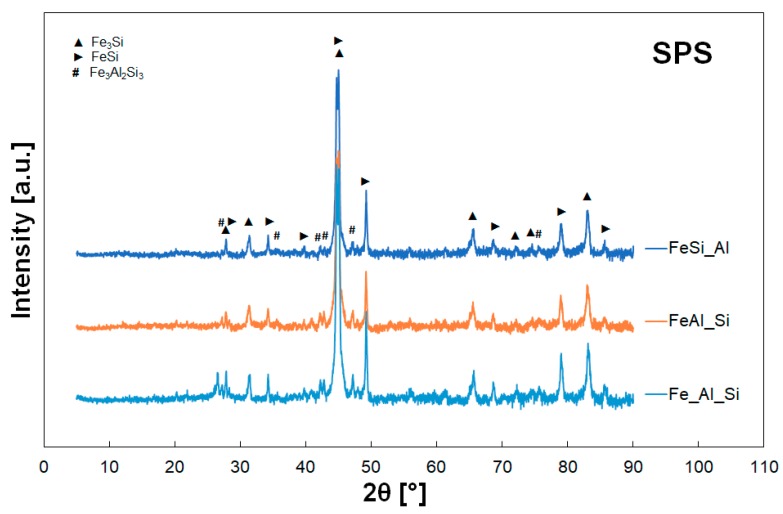
Phase composition of the sintered samples.

XRD patterns are in Figure 10 and they show only small changes compared to the mechanically alloyed powders. The XRD revealed that the sintered materials were predominantly composed of FeSi and Fe_3_Si phases (supersaturated by Al). Contrary to the MA powders the peaks of ternary phase Fe_3_Al_2_Si_3_ were identified in the sintered samples (compare Figure 7 and Figure 10).

Hardness measured for maximum load of 2 mN is represented in Figure 8 by solid horizontal line (dotted horizontal lines show the scatter of the data). The measured hardness was about 15–16 GPa and it was in good agreement with the values reached by the mechanically alloyed powders after 8 h of milling.

**Figure 11:**
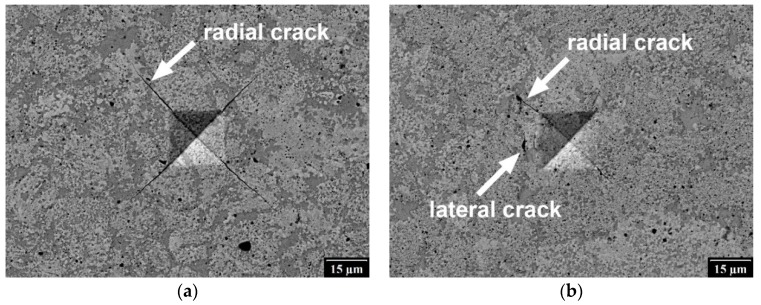
Cracks propagating from the Vickers indentation: (**a**) Fe_Al_Si, (**b**) FeAl_Si.

The fracture toughness was determined by measuring hardness, Young’s modulus and crack dimensions formed by the Vickers indentation to the maximum load of 5 N. The results are summarized in Table 1 and observed crack systems are shown in Figure 11. The measured hardness was about 13 GPa and Young’s modulus 225 GPa. Fracture toughness calculated using Equation (1) from measured radial (Palmqvist) cracks was about 3 MPa∙m^1/2^. It was lowest for the Fe_Al_Si sample reaching only 2.57 MPa∙m^1/2^. The highest fracture toughness was found for the sample FeAl_Si (3.61 MPa∙m^1/2^). The SEM images confirmed that the cracks were not connected under the indent, which means that they were radial (not median) cracks. The system of the radial cracks was well developed for the samples Fe_Al_Si and Fe_AlSi. For the samples FeSi_Al and FeAl_Si, the systems with only three cracks were also sometimes observed. These indents were excluded from the analysis and only the systems with four cracks were analyzed. On the other hand, lateral cracks were occasionally observed for the samples FeSi_Al and FeAl_Si with higher fracture toughness, which could be the reason for the increase in *K_IC_* (change of the mechanism of cracking associated with plastic deformation).

## 4. Discussion

The results presented in the previous section show that the kinetics of the mechanical alloying process depend on the initial powders which were mixed to obtain final homogeneous product. It can be stated that the microstructure, phase composition, hardness and Young’s modulus at the end of the mechanical alloying and after the spark plasma sintering are very similar. However, the kinetics of the formation of the homogeneous powder is different. All the measurements show that the fastest homogenization is for the Fe_Al_Si mixture made of pure elements. Full homogenization is reached after 4 h of mechanical alloying. 6 h are sufficient to homogenize the powders Fe_AlSi and FeSi_Al. The longest time (8 h) is required for the preparation of FeAl_Si mixture.

These differences are caused by the properties of the initial powders. For fast homogenization, it is advantageous to combine hard brittle and soft ductile powders. Ductile particles are easily plastically deformed and create cold welds. Brittle particles fracture, they get smaller and they can easily get inside soft plastic particles [11]. Only a small amount of the brittle hard particles causes fast increase in the hardness and accelerates the homogenization. This is the case for Fe_Al_Si and Fe_AlSi mixtures, in which Si has the role of the hard and brittle material in soft ductile Fe, Al, or AlSi30 pre-alloyed powder, respectively. Very fast increase in hardness of Fe_AlSi alloy during the first two hours of mechanical alloying can be caused by Si fine dispersion in the Al-Si eutectic. Increased temperature during mechanical alloying together with the fine Si eutectic particles in Al matrix accelerates the reactions with Fe and the formation of solid solution at the beginning of mechanical alloying. At the later stages of mechanical alloying, the primary Si acts similarly as the pure Si powder and the kinetics of the MA is equivalent with the case of the mixture of pure elements.

For the mixture FeSi_Al, the dominant component is hard FeSi25 intermetallic alloy. During the first two hours, the predominant hard particles are mixed with soft aluminum which causes the decrease in the hardness. Hardening processes are not fast enough to compensate this decrease caused by initial mixing. After 2 h of milling, homogenization occurs, hardness increases and the process continues in the same way as for Fe_Al_Si and Fe_AlSi mixtures.

The case of FeAl_Si mixture is fundamentally different because two hard brittle components are mixed. The brittle particles are preferentially crushed during the collisions and the formation of cold welds is more complicated. The diffusion between the components and the plastic deformation is lower and the homogenization takes a longer time.

As it was mentioned above, the particle size is slightly changing with milling time (from 30 to about 10 µm on Fe_Al_Si [17]). Only the particles larger than 10 µm were tested to avoid the effect of the particle size in soft surrounding matrix on hardness measurements. No substantial effect of particle size on hardness values was however noted, which is in agreement with the values measured on sintered samples (where the whole particle size distribution intervenes). Thus, the particles size distribution should not have a significant effect on the presented values of the hardness. As a result, the changes in hardness should correspond only to the changes in microstructure and homogenization of the powder particles caused by mechanical alloying.

Increase in hardness and the saturation on the constant value during mechanical alloying is generally observed [27,28]. The hardening rate depends on the kinetic energy of the collisions which can be driven by the milling parameters such as rotational speed, powder to ball ratio (material of the balls), etc. The lower rotational speed or lighter balls cause slower increase in hardness and require more time for the completion of the MA process [27]. The increase in hardness of the MA powders can be caused by different mechanisms. The first is work hardening when the particles are plastically deformed during the collisions with balls causing the increase of dislocation density [29]. The second mechanism is the grain refinement (Hall-Petch hardening) [28,30]. The next strengthening mechanism is dispersion hardening where the nanoparticles blocking the dislocation movement are homogenously distributed in the matrix. This type of hardening is typical for oxide dispersion strengthened steels [12]. The last type is the solid solution hardening where the atoms from the crystal structure are replaced by other atoms with not exactly the same atomic radius, causing the distortion of the lattice (the case of Al and Si atoms in the studied material). All these mechanisms can cause the increase in hardness observed in this study. When the equilibrium with the recovery mechanisms is established, the hardness does not increase more and saturates at the final level.

As it was discussed in previous paragraphs, the kinetics of the mechanical alloying and thus the strengthening mechanisms depend on the initial combination of the powders. If at least one component is ductile, the hardening mechanisms based on the plastic deformation and dislocation movement are easier and the hardening is faster. Increasing the amount of hard brittle components causes cracking and the particles cannot be easily plastically deformed. Thus, the hardening and the final homogenization is more time-demanding.

After the MA, FeSi and Fe_3_Si are predominant phases in homogeneous powders instead of the stable ternary phases [31]. It is caused by the easy contact of two particles during the milling and increased solubility of the elements caused by mechanical alloying [32]. Other phases found for the alloys with different Al/Si ratio (e.g., Fe_2_Al_5_ [17]) were not identified. Spark plasma sintering was chosen for the consolidation of the mechanically alloyed powders because it is a fast method which prevents significant changes in microstructure, grain growth and mechanical properties. Nevertheless, the presence of stable ternary phase Fe_3_Al_2_Si_3_ was observed after the sintering in all the samples. Exact phase ratio for Fe_Al_Si mixture was determined in the previous study [19] (33.0 wt.% of Fe_3_Si, 37.5 wt.% of FeSi, 29.5 wt.% of Fe_3_Al_2_Si_3_) and according to the diffractograms in Figure 10, there is no significant difference between various mixtures. This ternary phase usually complicates the processing of this type of materials by conventional methods because of its low plasticity. It would therefore be desirable to prevent this phase as well as the amorphous brittle phases before final processing. Compared to other initial compositions or processing ways (self-propagating high-temperature synthesis), the presence of the phases Fe_2_Al_5_ [8] or FeAl_2_Si [9], respectively, was not observed.

As can be seen from Figure 8, hardness after mechanical alloying was not affected by the sintering process and it stays very high (15 GPa). Hardness of compacted samples measured for maximum load of 5 N is slightly lower than the value measured for the load of 2 mN. This can be caused by several reasons which could include porosity of the sample (even if it was very low), cracking at higher loads or indentation size effect that could already be present at used low loads [33].

A wide range of formulas for different crack systems and materials exists for the determination of the fracture toughness by indentation methods (e.g., [34,35,36,37,38,39]). It was confirmed by SEM observations that the system of cracks in the studied material is radial (Palmqvist cracks). For this reason, Equation (1) which is valid in the range of crack lengths 0.25 ≤ *l*/*a* ≤ 2.5 was employed. The fracture toughness is quite low, in the range of ceramics [40], which could be caused by the high amount of stored plastic deformation and/or the presence of brittle amorphous phases. On the other hand, the manufacturing process performed in this study (i.e., mechanical alloying and spark plasma sintering) leads to significant fracture toughness improvement compared to standard casting of these materials (about 10 times [19]). The lowest fracture toughness was found for the mixture Fe_Al_Si which was homogenized in the shortest time. The highest fracture toughness was measured for FeAl_Si mixture, for which the homogenization took the longest time of 8 h. This means that the fast homogenization leads to a decrease of the fracture toughness. It can be caused by the stored plastic deformation in the powder particles. When the homogenization process is finished soon (e.g., after 4 h), no significant changes happen during further milling and only plastic deformation is stored in the particles. This means that from the point of the fracture toughness, the reduction of the milling time to the shortest time necessary for the homogenization of the powder would be optimal.

Other reasons for the differences in fracture toughness could be different levels of supersaturation of Fe_3_Si and/or FeSi phases by aluminum or different volume fraction of amorphous phase. All aluminum in solid solution in FeAl pre-alloyed powders could suppress the local liquid phase sintering. However, the TEM observation does not allow the exact quantification of the amorphous phase content in different pre-alloyed powders. On the other hand, no correlation between fracture toughness and grain size could be found as the grain size is practically the same for all pre-alloyed powders, which is probably closely connected with the saturation of the plastic deformation during milling.

## 5. Conclusions

The presented results show the differences in the kinetics of the mechanical alloying process depending on the initial feedstock materials used for the production of FeAl20Si20 alloy. The results can be summarized as follows:

- The final fine microstructure and measured mechanical properties of the powder mixtures are not significantly different. They are composed mainly by Fe_3_Si and FeSi phases supersaturated by Al.
- The mixture of hard (brittle) and soft (ductile) powders leads to shorter milling times, the mixture of hard initial powders requires longer milling times for complete homogenization.
- For the optimization of the production process (time for complete homogenization), the most efficient is the mixture Fe_Al_Si, followed by Fe_AlSi and FeSi_Al. Longest time to prepare homogenous powder is needed for FeAl_Si mixture.
- Fracture toughness is improved when the milling process is stopped immediately after the complete homogenization (highest fracture toughness was observed for the mixture FeAl_Si with the longest homogenization time).

## Figures and Tables

**Table 1:** Average grain size and fracture toughness of the samples prepared by SPS (measured at the maximum load of 5 N).

Powder Mixture	Grain Size [µm]	*H* [GPa]	*E* [GPa]	*a* [µm]	*l* [µm]	l/*a* [-]	*K_IC_* [MPa·m^1/2^]		
**Fe_Al_Si**	1.04	13.48	233.55	14.29	18.22	1.28	2.57	±	0.18
**FeAl_Si**	1.07	13.46	220.45	14.33	7.77	0.54	3.61	±	0.32
**Fe_AlSi**	0.94	13.37	231.03	14.50	16.53	1.14	2.73	±	0.28
**FeSi_Al**	0.88	12.61	220.66	14.26	9.95	0.70	3.21	±	0.40
